# Eye donation in hospice and hospital palliative care settings: perceptions, practice, and service development needs – findings from a national survey

**DOI:** 10.1186/s12904-023-01300-7

**Published:** 2023-11-08

**Authors:** Tracy Long-Sutehall, Anna Zatorska, Michelle Myall, Christina Faull, Adam Hurlow, Sarah Mollart, Clare Rayment, Jill Short, Jane Wale, Emma Winstanley, Mike Bracher

**Affiliations:** 1 School of Health Sciences, University of Southampton, Southampton, UK; 2 York and Scarborough Teaching Hospital NHS Foundation Trust, York, UK; 3 LOROS Hospice, Leicester, UK; 4 Leeds Teaching Hospitals NHS Trust, Leeds, UK; 5 West Suffolk Hospital NHS Foundation Trust, Bury St Edmunds, UK; 6 Marie Curie Hospice, Bradford, UK; 7 Rowans Hospice, Waterlooville, UK; 8 Milton Keynes University Hospital NHS Foundation Trust, Milton Keynes, UK; 9 National Health Service Blood and Transplant Services- Organ and Tissue Donation and Transplantation (NHS BT – OTDT), Speke, UK

**Keywords:** Corneal donation, Eye donation, Tissue donation, Behaviour change, Health services research, End of life care, Palliative care, Hospice care, Advanced care planning

## Abstract

**Objectives:**

New routes for supply of eye tissue are needed in the UK to support transplant surgery and medical research. Hospice care (HC) and Hospital-based Palliative care (HPC) services represent potential supply routes. This paper reports findings from the survey arm of the Eye Donation from Palliative and Hospice Care–Investigating potential, practice preference and perceptions study (EDiPPPP), objectives of which were to: i) Investigate existing practice in relation to eye donation across HC and HPC settings; ii) identify perceptions of HCPs toward embedding eye donation into routine end of life care planning; iii) investigate the informational, training, or support needs of clinicians regarding eye donation.

**Design:**

Online survey of UK-based HC and HPC clinicians, distributed through professional organisations (Association of Palliative Medicine (UK); Hospice UK).

**Participants:**

One hundred fifty-six participants completed (63% HC; 37% HPC—8% response rate, of *n* = 1894 approached).

**Results:**

Majority of participants (63%, *n* = 99) supported raising eye donation (ED) with patients and families and agreed that ED should be discussed routinely with eligible patients. However, 72%, (*n* = 95) indicated that staff within their clinical setting did not routinely discuss the option of ED in end-of-life care planning conversations with the majority of participants reporting that the option of ED was not ‘routinely discussed in multi-disciplinary team or other meetings.

**Conclusions:**

Despite significant support, ED is not part of routine practice. Attention to barriers to embedding ED and reducing knowledge deficits are urgently needed to increase the supply of eye tissue for use in transplant operations.

**Supplementary Information:**

The online version contains supplementary material available at 10.1186/s12904-023-01300-7.

## Background

Globally, the estimated number of people who are visually impaired is reported (by WHO databases) to be 285 million, with 39 million individuals recorded as blind, and 246 million recorded as having low vision [1]. In the UK, the Royal National Institute of Blind (RNIB) reports that over two million people live with sight loss (predicted to double to nearly four million by 2050) [2] with around 5000 corneal transplants needed annually in the UK [3]. Significantly, the actual number of people waiting for a corneal transplant is difficult to confirm, as there is no centralised waiting list for patients who need a corneal transplant (unlike solid organ donation). A further pressure on the supply of eye tissue is that approximately 30% or retrieved tissue will be discarded due to infection/viruses, with supply further compromised by a 28-day limit to storage requiring disposal of tissue thereafter [4, 5].

Diseases leading to sight loss can be treated if eye tissue is available, tissue which is only available via Eye Donation (ED) [2]. However, the supply of eye tissue in the UK does not currently meet demand. The National Health Services Blood and Transplant (NHS BT) Tissue and Eye Services (TES) Bank in Speke, Liverpool (who supply most eyes for UK surgery) aim to achieve a weekly stock of 350 eyes so that they can provide 70 eyes every working day for treatment or research. From April 2021 – March 2022 donation of eyes from all sources (solid organ donation, tissue donation) generated 4,555 eyes from 2,286 donors equating to only 13 eyes per day and 88 eyes available per week.

One underexplored potential supply route is from patients dying in Hospice Care (HC) and Hospital-based Palliative Care (HPC), where ED may be an option for many as organ and other tissue donation is typically not. Our recent review of case notes from six services in England against NHSBT criteria for referral for ED indicated that 46% of patients in three HPC and 56% in three HC settings met the eligibility criteria to be an eye donor [6], however across the 553 patients who met referral criteria, only 14 cases (3%) had been referred to NHSBT.

Health Care Professionals (HCPs) function as gatekeepers to raising (or not) ED in care planning conversations; providing (or not) ED-related information; and facilitating (or not) referral to retrieval services if ED is to be realised. Therefore, in designing interventions to realise the potential for ED from HC and HPC settings, national organisations steering practice and process need to be aware of the context in which ED related behaviours take place, identifying barriers (attitudinal, behavioural, organisational, and/or resource related) which may exist. This paper reports findings from an online survey of HCPs in UK-based HC and HPC services, undertaken as part of the NIHR funded EDiPPPP study [7].

Survey objectives were to:

I. Investigate existing *practice* in relation to ED across HC and HPC settings;
II. Identify *perceptions* of HCPs of embedding ED into routine end of life care (EoLC) planning;
III. Investigate the informational, training, or support needs of HCPs needed to embed ED in routine practice.

## Methods

### Survey development

Survey questions were developed following: i) secondary analysis of published and unpublished data shared with the research team by clinical co-applicants (SG, JW, CR, SM) [8, 9] ii) search of global literature [10]; iii) analysis of interviews and focus group data undertaken in Work-Package 1 of EDiPPPP [7]. Questions were grouped into domains comprising the EDiPPPP study’s foci (*practice*, *preferences*, and *perceptions.*

Survey drafts were produced (MJB, MM, TLS) and piloted with clinical co-applicants (CR, JS). The final instrument comprised 61 questions (see Supplementary file 1) using the Microsoft Forms online survey system. Surveys were completed anonymously, and respondents were requested not to provide information that could potentially identify themselves, patients, or carers.

### Recruitment strategy

The *Association for Palliative Medicine (UK)* (*n* = 1222 approached), and *Hospice UK* (*n* = 672 approached) distributed the survey to their members via email in week three of November of 2020, with a two-week follow up email in week one of December 2020. The survey closed on 31^st^ December 2020.

### Data analysis

Data from closed questions generated descriptive statistics (e.g. percentages of respondents providing a given answer; average completion time for survey), while data from free-text responses underwent qualitative content analysis [11]. Both analytic strategies were undertaken using Microsoft Excel. Content analysis of qualitative data involves identification of analytically relevant content, and organisation of these observations into categories of interest through the application of codes. Both inductive codes (as derived from observations of the data) and deductive codes (applied from a pre-determined framework such as a theory, or observation schedule) [11] were applied in analysis of the free text commentary survey responses.

## Results

### Response rate and completion time

The survey was completed by 156 participants, representing an 8% response rate (of *n *= 1894 approached); 63% (*n* = 98) reported working in HC settings, with 37% (*n* = 58) reporting HPC. The initial invitation generated 103 responses, with a further 53 after follow-up. Median completion time was 16 min and 12 s (IQR = 10:28 – 23:46).

### Sample demographics

**Fig. 1:**
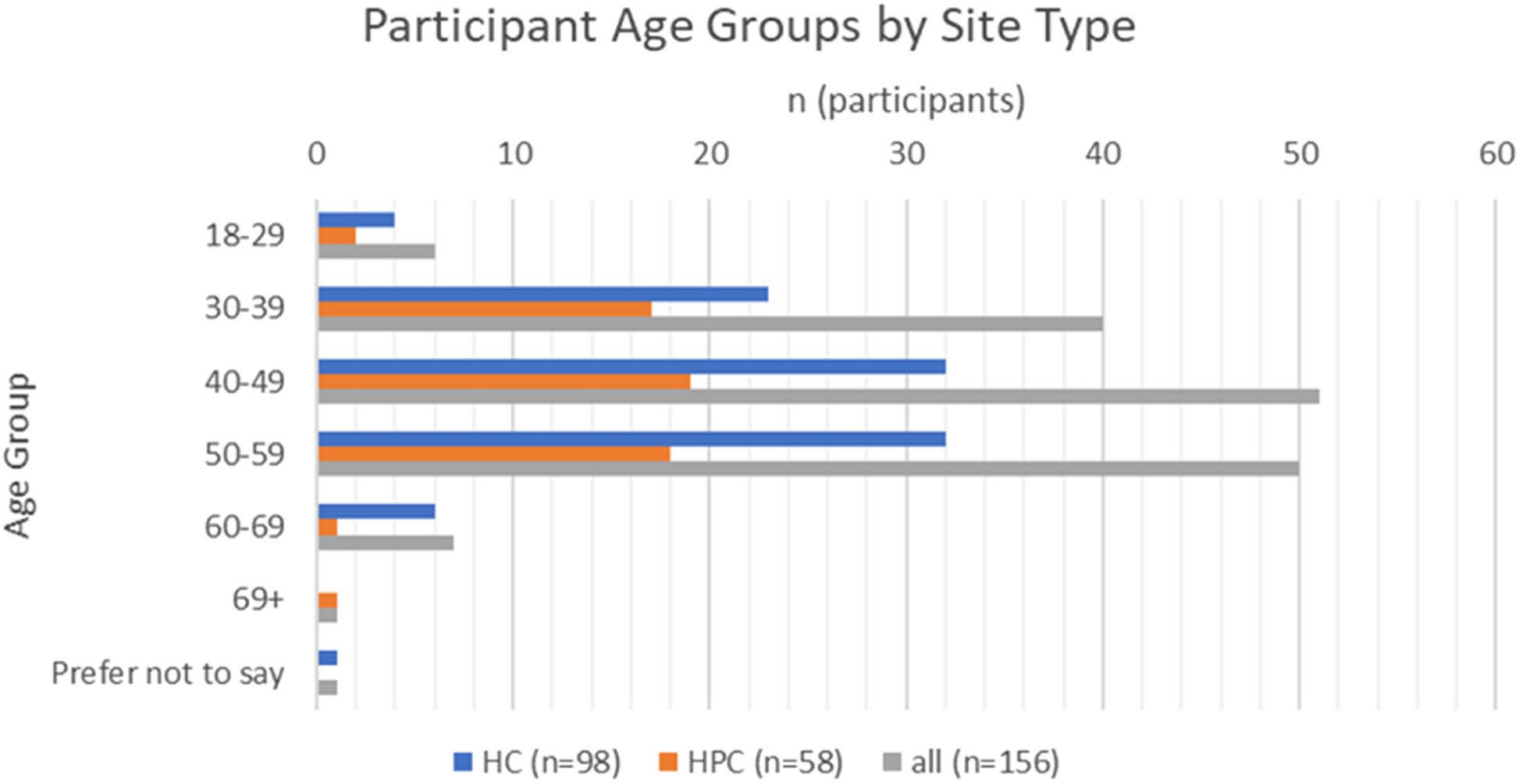
National survey participant age groups by site type

For all participants: 65% (*n* = 101) were aged between 40–59 years, with 30% (*n* = 46) aged 18–39 (Fig. 1). Eighty-three percent (n = 129) identified as female; and 87% (*n* = 136) as white British ethnicity. Demographic characteristics were similar for both HC and HPC groups.

**Fig. 2:**
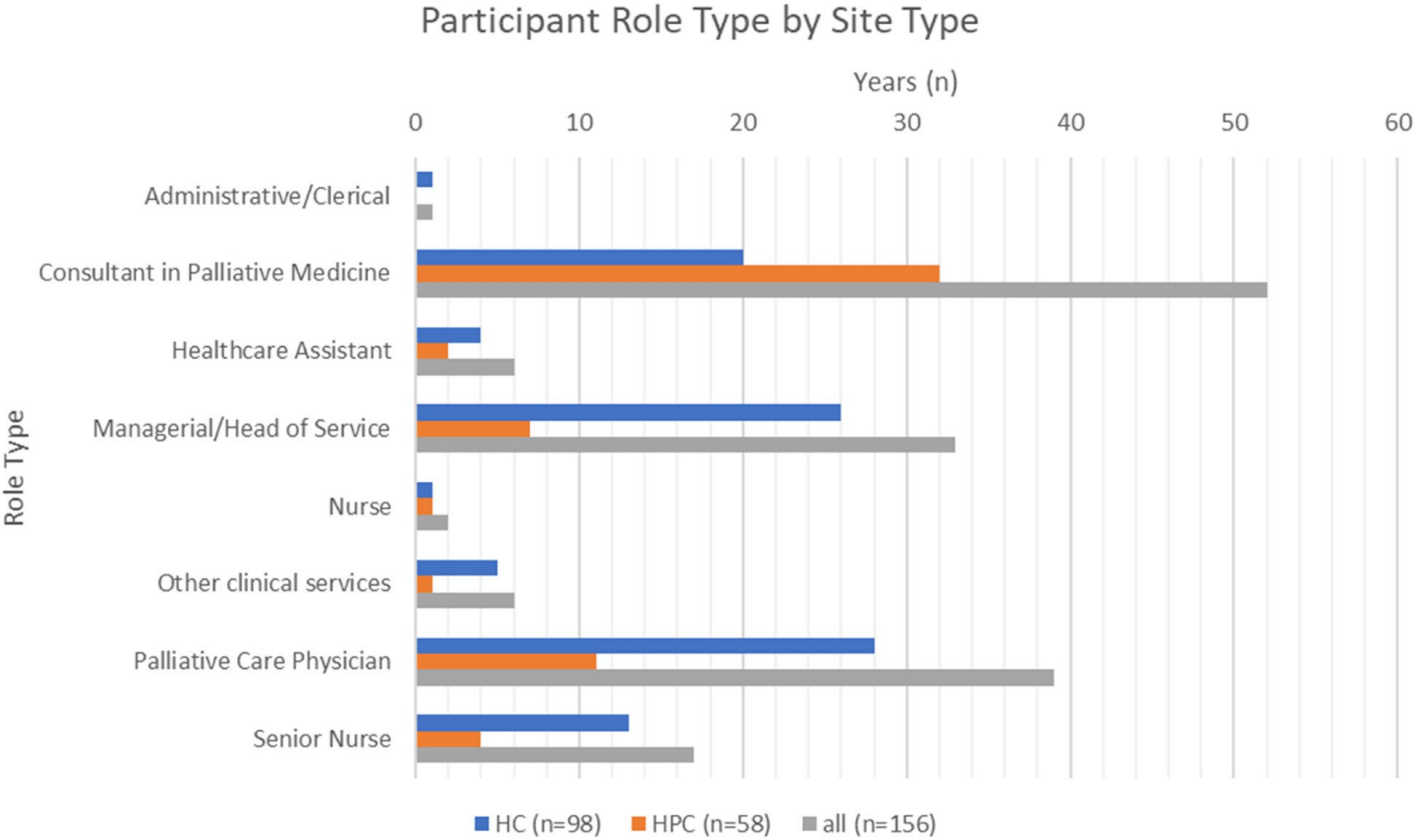
Participant demographics – role

Respondents identified as: Consultant in Palliative Medicine 33% (*n* = 52), Palliative Physician 25% (*n* = 39), Managerial/Head of Service role 21% (*n* = 33), Senior Nurse 11% (*n* = 17) with 6% indicating Other clinical services (e.g. Counselling, Physiotherapy, Advance Care Planning Facilitator, Specialist Pharmacist), Healthcare Assistants 4% (*n* = 6); Nurses (excluding senior roles 1% (*n* = 2) and Administrative/Clerical role 1% (*n* = 1) (Fig. 2).

## Findings

### Objective I: investigate existing routine practice in relation to ED across HC and HPC settings

#### Current practice in raising and discussing the topic of ED with patients and families

Questions sought participant experiences of ED-related practice (Table 1) Eighty-four percent of participants (*n* = 131) indicated that they were ‘[a]ware that ED is an option that patients can choose as part of advance care/end of life care planning’, with 15% (*n* = 24) indicating that they were not aware that ED was an option for patients in this clinical setting.

**Table 1:**
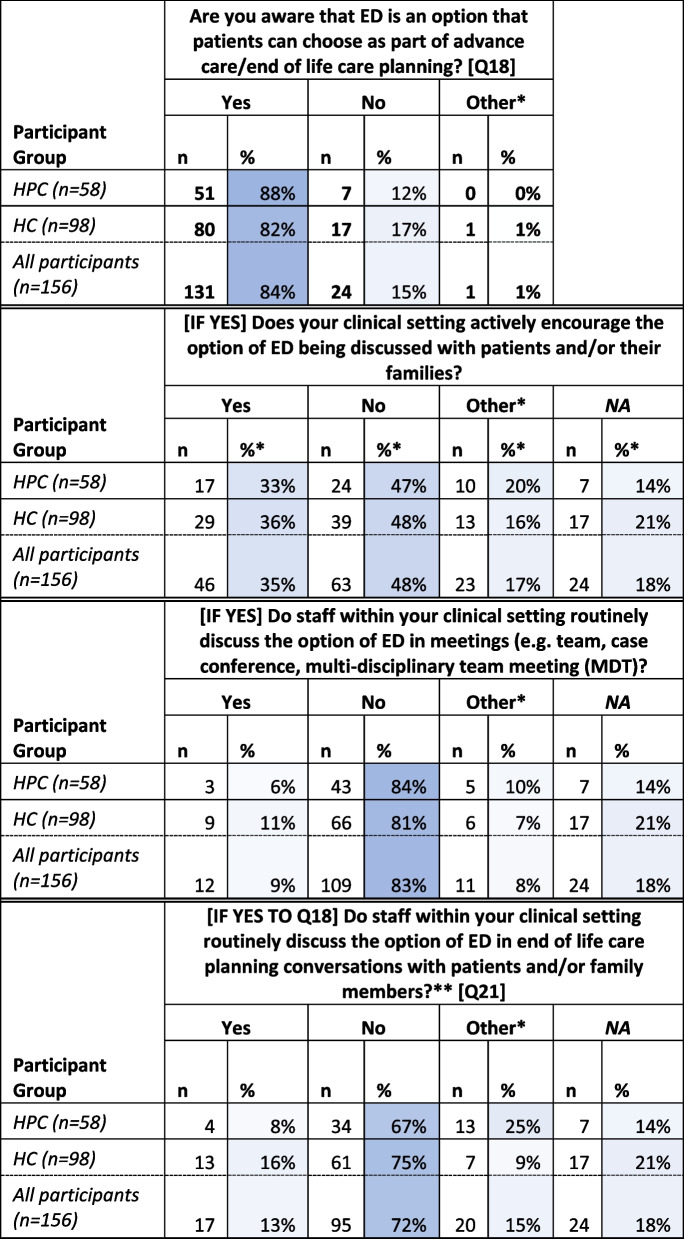
Responses to questions relating to ED practice (Q18-22; 35 – greater intensity of blue indicates value closer to 100%)

^*^''Other' indicates that this option was selected, and a free-text response was provided

^**^NA indicates 'Not applicable' due to a negative answer to Q18—row percentages for all questions marked with a ** in this table are calculated using the number of participants for each role type who completed each question (i.e. the total number for each role type minus the number of NAs)

Of those aware that ED was an option, a majority (48%, *n* = 63) indicated that their clinical setting does not ‘actively encourage the option of ED being discussed with patients and/or their families’ with 35% (*n* = 46) indicating that their setting did. Only fifteen participants selecting ‘Other’ provided comment indicating that ED was included in some form of organisation documentation but that clinicians may make an active decision not to discuss the option based on their assessment:

*“It is mentioned on the admission paperwork as an option to possibly discuss, it is rarely appropriate due to their underlying diagnosis of current clinical condition” (Palliative Physician, HC setting).*

A majority of respondents (72%, *n* = 95) indicated that staff within their clinical setting did not ‘routinely discuss the option of ED in end-of-life care planning conversations with patients and/or family members’, with the majority of participants who were aware that ED was an option (83%, *n* = 109) reporting that the option of ED was not ‘routinely discussed in multi-disciplinary team or other meeting’:

“*We try to make this the case, but in practice it doesn't always happen” (Consultant in Palliative Medicine, HC setting)*

In response to the question: ‘Which of the following best describes your current practice?’, 44% (*n* = 68) of total participants indicated that they discuss ED ‘only when the subject is raised by patients or families’ with 22% indicating that they ‘never discuss ED with patients or families’. Only 13% (*n* = 20) of total participants indicated that they ‘routinely discuss [this option] with patients and families’. Results also indicate that the majority of participants had not raised the option of ED in the past year (Table 2).

**Table 2:**
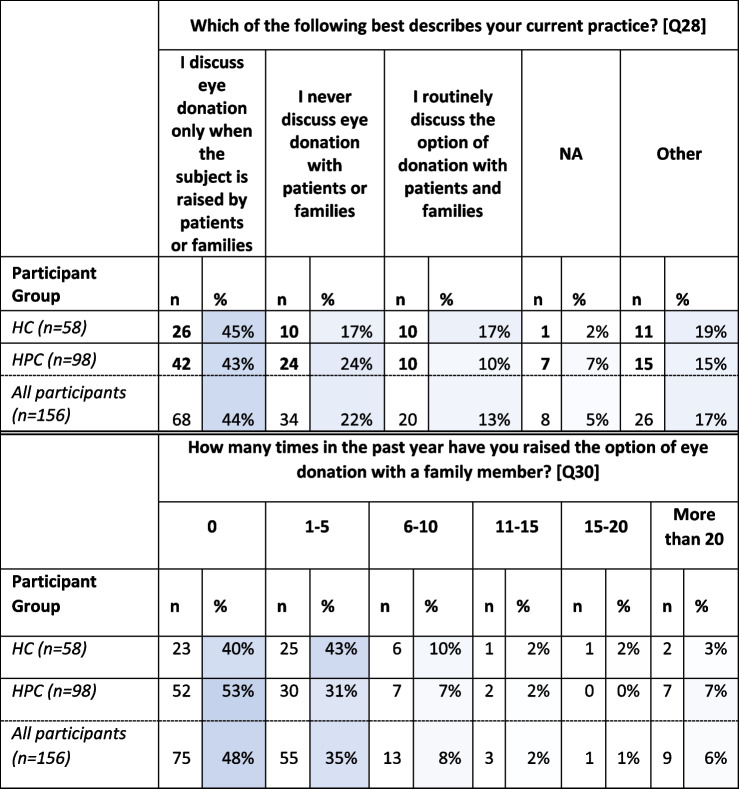
Responses to questions regarding to discussing ED with patients Q28, 30- greater intensity of blue indicates closer to 100%)

The following findings are limited to those participants who indicated that they had experience of discussing ED with patients or family members (*n* = 115). Eighty-eight percent (*n* = 101) of these participants had discussed ED with family pre-death of a patient, with 20% (*n* = 23) reporting post-death discussions with family.

### Objective II: identify the perceptions of healthcare staff of embedding ED into routine end of life care planning.

#### Perceptions of HCPs regarding propriety and feasibility of discussing ED with patients and/or families

Participants were asked to indicate their response (i.e. ‘Agree’, ‘Unsure’, or ‘Disagree’) in relation to a series of statements regarding the option of ED being raised with patients and families. Seventy-three percent (*n* = 114) of total participants *disagreed* with the statement ‘[d]iscussing ED is too distressing for a patient and/or their family’ with sixty-three percent (*n* = 99) of total participants agreeing that ED *should be discussed with* eligible patients and/or their families. Sixty-one percent (*n* = 95) of total participants *agreed* that they ‘feel confident in starting a conversation about ED with a patient and/or their family’, however half of respondents (50%, *n* = 78) indicated that they had some concerns about how a patient or family might respond (Table 3).

**Table 3:**
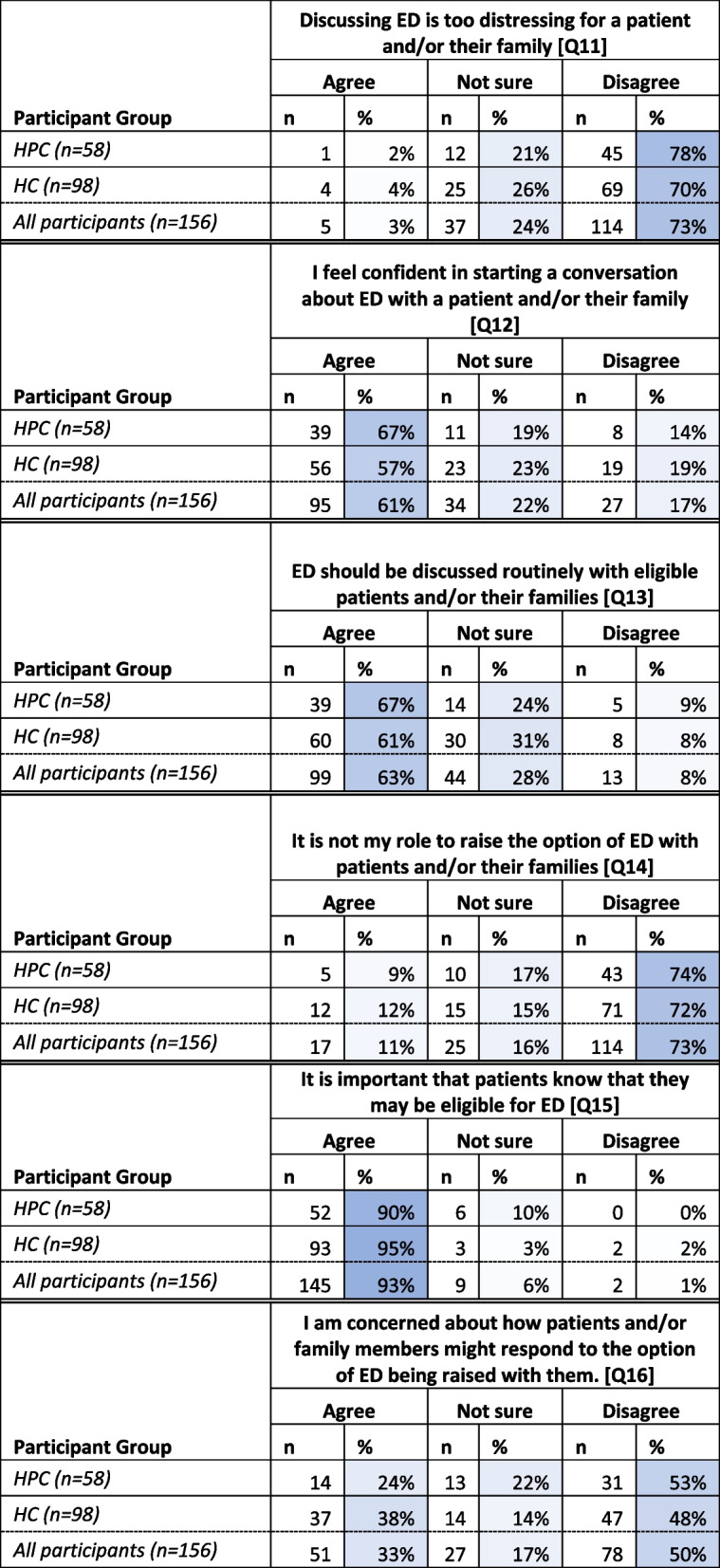
Responses to statements relating to participant perceptions of eye donation (Q11-16—greater intensity of blue indicates closer to 100%)

The majority of participants indicated that it was their role to raise the option of ED (73%, *n* = 114) and that it was important that patients know that ED is an option for them if it is (95%, (*n* = 145). (Table 3).

Participants were asked about *when* the option of ED should be raised with patients with the majority of participants indicating that this discussion should take place ‘during advanced care planning (80%, *n* = 125), which could take place at multiple time points across the end-of-life care planning trajectory.

#### Availability of clinical guidance and information to support ED conversations with patients/families

Sixty-one percent (*n* = 81) indicated that their setting does not ‘include ED in its admission documentation’ (Table 4). Forty-six percent (*n* = 61) of participants who were aware that ED was an option for patients indicated that their clinical setting did not ‘have clinical guidelines in written form that include ED*’*, and fifty-eight percent (*n* = 90) of total participants indicated that there was no ‘donation link person or champion in [their] service’ with 24% (*n* = 38) responding ‘[d]on’t know’. Sixty-one percent (*n* = 81) indicated that written information for patients and family was available in their clinical setting (Table 4).

**Table 4:**
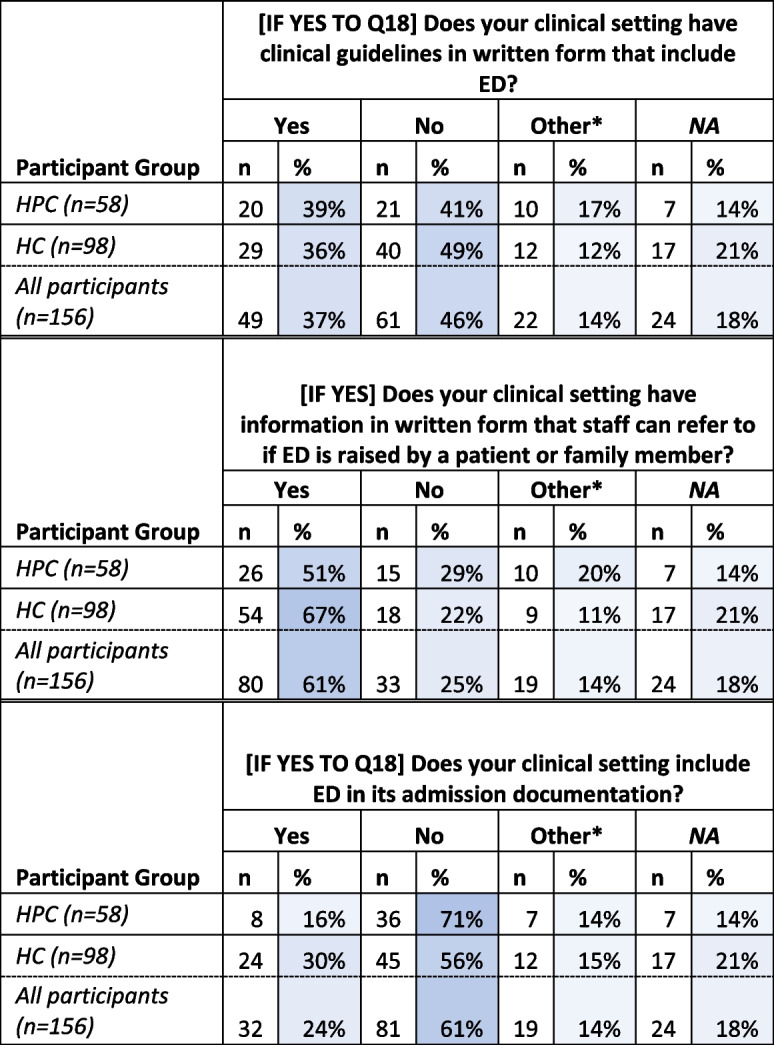
Responses to statements relating to participant experiences of availability of clinical guidance and information to support eye donation conversations with patients/families (Q24-26 - greater intensity of blue indicates closer to 100%)

^*^''Other' indicates that this option was selected and a free-text response was provided

^**^NA indicates ‘Not applicable’ due to a negative answer to Q18—row percentages for all questions marked with a ** in this table are calculated using the number of participants for each group who completed each question (i.e., the total number for each participant group minus the number of NAs)

### Objective III: investigate the existing informational, training, or support needs of HCPs needed to embed ED in routine practice

#### Knowledge of contraindications and processes for ED

To assess HCPs’ current knowledge base regarding ED we included a short section ‘flash quiz’, exploring three key questions about the ED pathway. Content analysis was performed on the free-text responses to categorise them by type (Table 5). Responses to the question ‘how long after death can eye donation take place?’ indicated that just over half of all participants (53%, *n* = 83 of 156) understood that ED can take place up to 24 h after death. Eighteen percent of participants (*n* = 28 of 156) indicated that they did not know the time limit.

**Table 5:**
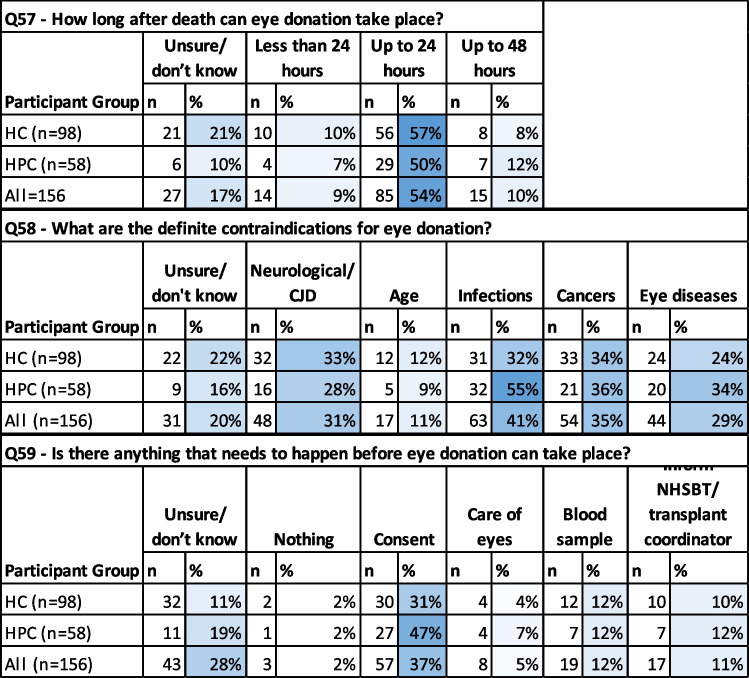
Flash quiz questions

When asked to identify definite contraindications for ED (in free-text responses), infections were most commonly indicated (41%, *n* = 63), followed by cancers (35% *n* = 54), and neurological conditions (31%, *n* = 48). When asked what should happen before ED can take place, 37% (*n* = 57) participants indicated that patient or family consent was required (this is correct), while 12% (*n* = 19) indicated that a blood sample is required (this is correct).

#### Training and resource to support ED

Most respondents had not received any training about ED (61%, *n* = 95), while for those who had received training, the training had been provided by their clinical site hospital or hospice (44%, *n* = 61) or other sources (56%, *n* = 34). Fifty-four percent of participants who had training undertook this at least 24 months prior to completion of the survey. Seventy-two percent of all participants completing a training programme (*n* = 61) stated that it *did* provide them with the information they needed to be confident in discussing eye donation with patients/family (Table 6).

**Table 6:**
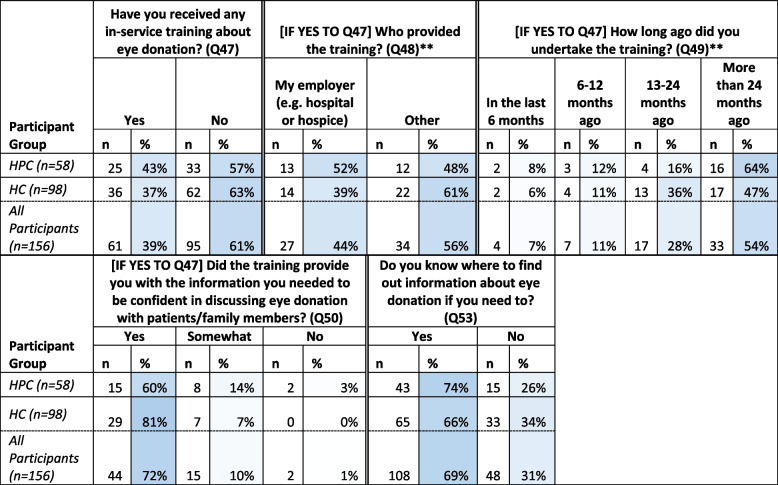
Results from survey questions exploring previous ED-related training

^**^Row percentages for all questions marked with a ** in this table are calculated using the number of participants for each group who completed each question (i.e. the total number for each participant group minus the number of NAs)

To gain some insight into the content and quality of the training participants were asked to comment on the positive or negative aspects of the training they received. Many comments indicated an initial positive impact of the training, together with concern about longer term embedding of this in routine practice:

“It has a brief effect on practice and then it fades!” (Consultant in Palliative Medicine, HPC setting)

“Everyone very enthusiastic but it has not been effectively implemented” (Manager/Head of Service, HPC setting)

Participants providing free-text responses indicated several ‘unmet knowledge or training needs’ including: the eligibility criteria for ED; processes needed to facilitate ED; and best practice in communication with patients and families:

“I would appreciate an update in the new legislation and local procedures and guidance on contraindications/ which organs and how to go about it.” (Consultant in Palliative Medicine, HPC setting)

"I really don’t know what I don’t know. I am rusty on how to start the conversation, how to instruct a patient, what to say to the family about the process, the eligibility criteria, the register etc" (Manager/Head of Service, HPC setting)

## Discussion

Findings from this survey indicate a paradoxical stance toward ED in hospice and palliative care settings. Participants demonstrated positive attitudes toward ED, but despite this ED is not routinely offered to patients as part of end-of-life planning. Research applying social cognitive theories in donation contexts reminds us that there is no simple linear causal relationship between attitudes, values, willingness, and action related to donation behaviours [12]. Specifically, positive views toward ED are not sufficient to ensure this practice is undertaken, as indicated in this paper and previous single-site surveys on this topic [8, 13] as well as results of interviews with HCPs conducted as part of the EDiPPPP study (in press).

Despite responses indicating ‘knowledge’ that ED was an option that could be included in advanced care/end of life care planning, and agreement that it is important that patients know that ED is an option that they may be able to choose, a majority of participants indicated that staff within their setting did not routinely discuss the option of ED, nor was ED an agenda item for multi-disciplinary team meetings. Reluctance to raise the issue of ED with patients has been linked to HCPs views that discussing ED is ‘distressing’ for patients and family members [14, 15]. Extant literature report HCPs beliefs that discussing ED detracts from the tranquil environment of a hospice with donation requests potentially causing patients and their families physical and psychological harm [16]. However, in our survey most participants disagreed with the statement that ED was a distressing topic. Therefore, if ED was not perceived as a distressing topic by staff in these settings, and there is agreement that ED is an option of which patients should be aware, why is ED not embedded in routine practice (e.g. in Advanced Care Planning (ACP) conversations)?

Behaviour change theory may offer some useful perspectives on this issue. The COM–B Model of Behaviour [17] identifies key factors for implementing and embedding change in individual and organisational behaviour. The model proposes that specific behaviours occur only when an individual perceives that they have capability, opportunity, and are motivated to enact a specific behaviour rather than another. HCPs in our study indicate that they are motivated to offer the option of ED, and many also indicate capability; however, specific events and supporting resources (e.g. clinical guidance documents, care planning strategies) do not currently support the opportunity to enact this behaviour, meaning that HCPs may not be clear about when this option should be raised. Furthermore, there are evidenced knowledge gaps which may undermine responses to ‘triggers’ to open this topic. For example, while undertaking the EDiPPPP study, HCPs shared informally that whilst a donation question (a trigger) is included in the Recommended Summary Plan for Emergency Care and Treatment (ReSPECT) form, in their experience this question is often not completed. This is reflected in a recent single-site HC service improvement initiative which included a retrospective note review of ReSPeCT forms over a three month period [18]. Findings indicate that no forms were fully completed, and while ‘clinical recommendations’ and ‘resuscitation decisions’ had high levels of completion there were lower rates of completion for patient preferences, priorities of care and what is important to the patient.

Findings from the national survey clearly indicate knowledge deficits regarding the process of ED and the lack of standardised training related to ED available to HCPs. Evidence supports the link between HCPs education and increased rates of identification of potential donors and consent and donation rates with authors reporting that improving knowledge, e.g., perceptions of the benefits of donation and objective knowledge such as timelines, contraindications, process of referral [19] increases HCP confidence. Furthermore, knowledge and education about the donation process more broadly with specific focus on the questions that may be raised by patients and family members will facilitate early discussions with patients (pre death) so that patients can discuss the option with family members [20, 21]. We propose that education and training is fundamental to HCPs perceptions of their ‘capability’ in implementing ED into usual end of life care planning. As the majority of respondents indicated that they had not received any education and training, with those that had having done so at least 24 months previously, knowledge deficits may be demotivating HCPs to actively seek opportunities to raise the issue of ED with patients and family members.

To feel confident and competent in raising ED HCPs need access to clinical guidance and care planning documentation that stimulate the option of ED being raised as part of routine practice. Clinical guidance regarding the eligibility criteria for ED and process of referral of potential eye donors is currently missing from the resources provided to clinicians seeking to offer organ donation to family members by NHSBT. This lack is currently being addressed as an outcome of the EDiPPPP study (in press).

### Study limitations

We acknowledge the low response rate for this survey (8%) of the target population. We propose two factors that have impacted the response rate, I) as the survey period coincided with two consecutive lockdown periods across England during which significant demands were made on clinical staff, specifically HC and HPC settings [22] (as death rates increased) the effect of the COVID-19 pandemic was significant, II) the survey invitation entered a highly saturated communication environment (e.g. institutional communications relating to COVID, guidance from professional bodies, requests for evidence to support rapid evidence reviews relating to COVID). Despite the low response rate the fact that HCPs made time to respond and provide detailed open text comments, underpins the importance of this issue for clinicians keen to embed ED in practice.

## Conclusion

This study indicates that whilst individual HCPs may be supportive of the option of ED being raised with patients few settings have ‘mandated’ this practice or provided necessary guidance and resource for it to become part of routine practice, nor does it appear that patient preferences are routinely identified. There appear to be missed opportunities where the option of ED could be raised as part of ongoing care planning and these missed opportunities appear linked to a lack of institutional support and resources to support these conversations. As evidence indicates that HC and HPC settings represent significant potential routes of supply for eye tissue, without ED becoming an embedded practice, this potential will be unrealised, patient and family wishes for donation are likely to remain unfulfilled and those waiting for the eye tissue needed for sight saving and sight restoring surgery, will continue to wait and suffer the impact of sight loss on their, and their families, lives.

### Supplementary Information

**Additional file 1.**

## Data Availability

Please contact the corresponding author regarding availability of data.
