# Supplementary material for: Eye donation in hospice and hospital palliative care settings: perceptions, practice, and service development needs – findings from a national survey
Source: BMC Palliat Care. 2023 Nov 8;22:173. doi: 10.1186/s12904-023-01300-7 (PMC10631126; doi:10.1186/s12904-023-01300-7)
Supplement: Supplementary file 1 — Additional file 1. [file 12904_2023_1300_MOESM1_ESM.docx]

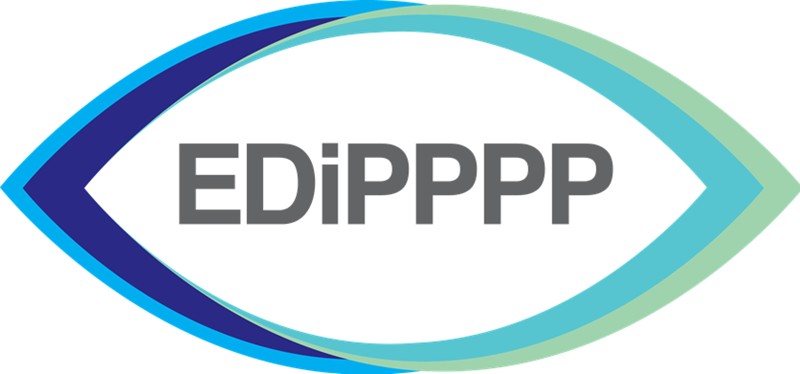


Eye Donation from Palliative and Hospice Care Contexts (EDiPPPP)

Health Care Professionals Online Survey


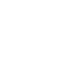


* Required

# Consent for participation

1. Please tick (check) this box to indicate that you consent to taking part in this survey.

*


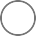
 I agree to participate in this survey

# Demographics

The following questions are about you and your role (note: please do not provide specific information such as the name of your hospital/trust/hospice, as this information could potentially be used to identify you)

1. What is your age? *


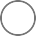
 18-29


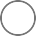
 30-39


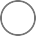
 40-49


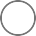
 50-59


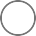
 60-69


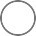
 69+


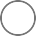
 Prefer not to say

1. Please indicate if you identify as: *


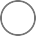
 Male


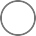
 Female


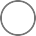
 Prefer not to say

1. Please indicate which ethnic group you most identify with *


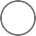
 White British


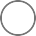
 White Irish


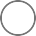
 Other white


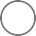
 White and black Caribbean


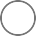
 White and Black African


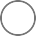
 White and Asian


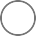
 Other Mixed


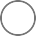
 Indian


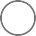
 Pakistani


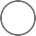
 Bangladeshi


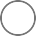
 Other Asian


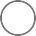
 Prefer not to say


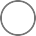


Other

1. Which clinical setting do you currently work in? (please DO NOT specify your institution, as this information could potentially be used to identify you) *


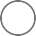
 Palliative Care


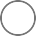
 Hospice Care


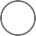


Other

1. What is your current role? (please state below but DO NOT specify your institution, as this information could potentially be used to identify you) *
2. How many years have you worked in palliative or hospice care settings? Please indicate number of years below *

# Perceptions

Questions in this section ask about your views toward organ and tissue donation in general, and eye donation in particular.

1. Are you currently registered to be an organ/tissue donor? *


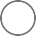
 Yes


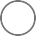
 No


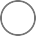
 Prefer not to say

1. Have you stipulated any organs and/or tissues that you do not want to donate? *


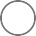
 Yes


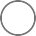
 No


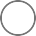
 Prefer not to say

1. If yes, please indicate below which organs or tissues you have indicated that you do not want to donate *

# The following questions are about how you feel about eye donation

Please indicate whether you agree or disagree with the following statements

1. Discussing eye donation is too distressing for a patient and/or their family *


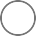
 Agree


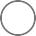
 Not sure


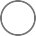
 Disagree

1. I feel confident in starting a conversation about eye donation with a patient and/or their family *


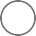
 Agree


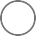
 Not sure


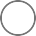
 Disagree

1. Eye donation should be discussed routinely with eligible patients and/or their families *


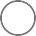
 Agree


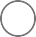
 Not sure


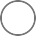
 Disagree

1. It is not my role to raise the option of eye donation with patients and/or their families *


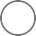
 Agree


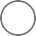
 Not sure


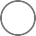
 Disagree

1. It is important that patients know that they may be eligible for eye donation *


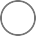
 Agree


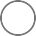
 Not sure


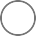
 Disagree

1. I am concerned about how patients and/or family members might respond to the option of eye donation being raised with them. *


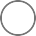
 Agree


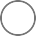
 Not sure


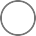
 Disagree

1. If you wish to add any comments regarding how you feel about the option of eye donation, please use the space below

# Practice

The following questions are about your knowledge and current practice relating to the option of eye donation as part of end of life care

1. Are you aware that eye donation is an option that patients can choose as part of advance care/end of life care planning? *


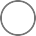
 Yes


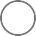
 No


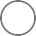


Other

1. Does your clinical setting actively encourage the option of eye donation being discussed with patients and/or their families? *


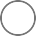
 Yes


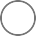
 No


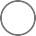


Other

1. Do staff within your clinical setting routinely discuss the option of eye donation in meetings (e.g. team, case conference, multi-disciplinary team meeting (MDT))? *


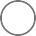
 Yes


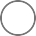
 No


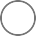


Other

1. Do staff within your clinical setting routinely discuss the option of eye donation in end of life care planning conversations with patients and/or family members? *


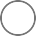
 Yes


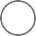
 No


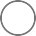


Other

1. Does your service routinely check the Organ Donor Register status of patients at the point of admission? *


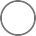
 Yes


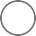
 No


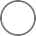


Other

1. Please summarise the process for checking Organ Donor Register status in your service (if unsure please write 'unsure') *
2. Does your clinical setting have clinical guidelines in written form that include eye donation? *


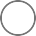
 Yes


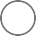
 No


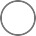


Other

1. Does your clinical setting have information in written form that staff can refer to if eye donation is raised by a patient or family member? *


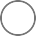
 Yes


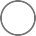
 No


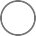


Other

1. Does your clinical setting include eye donation in its admission documentation? *


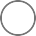
 Yes


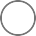
 No


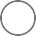


Other

1. Please comment below if you would like to share any thoughts on discussing the option of eye donation with patients and/or families.
2. Which of the following best describes your current practice? *


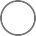
 I never discuss eye donation with patients or families


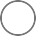
 I discuss eye donation only when the subject is raised by patients or families


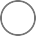
 I routinely discuss the option of donation with patients and families


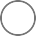


Other

1. At what point in a patient's care should the option of eye donation be discussed? (Please select all that apply). *


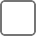
 Before admission


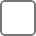
 During admission


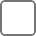
 At first assessment by palliative care services


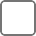
 During in patient stay


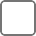
 During advanced care planning


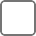
 Never


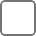


Other

1. How many times in the past year have you raised the option of eye donation with a family member? *


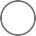
 0


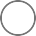
 1-5


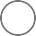
 6-10


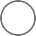
 11-15


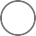
 15-20


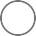
 More than 20

1. Reflecting back on the last time you discussed eye donation with a patient and/or relative, how did you feel having that conversation? *


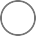
 I have never discussed eye donation with a patient or family member


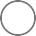
 Somewhat uncomfortable


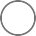
 Very uncomfortable


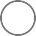
 Neither comfortable nor uncomfortable


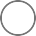
 Somewhat comfortable


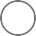
 Very comfortable

1. If you have had a discussion with a family member, was this discussion pre or post death of the patient? (please select all that apply) *

Pre-death

Post-death

I have not had a discussion with a family member

1. Who do you think should raise the issue of eye donation with a patient or family member? (Please select all that apply) *

It is my role

The GP should have this conversation

The Consultant referring the patient to Palliative Care

Other palliative care specialist (e.g. CNS)

A donation specialist

All of the above

Other

1. Please summarise why you think the people above should raise the issue of eye donation with a patient or family member. *
2. Is there a donation link person or champion in your service? *

Yes

No

Don't know

1. If yes, what is the position of the link person or champion in your service? (please describe their position, but DO NOT add personal information such as names or institutions)

# Legislation governing eye donation - knowledge and experience of practical changes

The next set of questions will ask you about recent changes to legislation governing organ and tissue donation in England.

This is commonly referred to as a move from an 'opt in' to an 'opt out' system (and also known as 'Max and Keira's Law').

Please answer these questions to the best of your knowledge, without undertaking additional reading or source checking (i.e. searching for answers online).

1. Please indicate your response to the following statements: *

True False Unsure

Patients in England are assumed to consent to

organ and tissue donation unless they

opt out.

In the case of a patient 18 years or older with mental capacity, family/next of kin (NoK) cannot refuse donation unless the patient had indicated their wish to opt out prior to death.

Patients can opt out of donating specific

organs or tissues while maintaining a wish to

donate others.

1. Please indicate your response to the following statements *

Strongly disagree

Somewhat disagree

Neither agree nor disagree

Somewhat

Agree Strongly agree

I am aware of the procedure for opting out of organ and tissue donation in England.

I am aware of the procedure for

specifying organs and tissues that I do/do not

wish to donate.

I am confident in my ability to talk through with patients and/or

families the necessary processes for opting

out of organ or tissue donation in England.

1. Please summarise your current understanding of the processes for opting out of organ or tissue donation, providing as much detail as you would to a patient or family member/NoK in your service (if unsure, please write 'unsure')
2. Please indicate your response to the following statements: *

Very negatively

Somewhat Negatively

Neither positively nor negatively

Somewhat

positively Very positively

Overall, the move to the opt out system has affected my clinical practice…

Overall, the move to an opt out system has

affected patients and families/NoK within my

service...

1. Following your answer to the question above, please describe below the impact that the move to an opt out system has had on your clinical practice, and/or the patients and families/NoK within your service.

(if none or if you are unsure, please write 'unsure' below)

1. Have you received any information regarding the move to opt out legislation? (e.g. from your institution, or from other bodies such as NHS Blood and Transplant) *

Yes

No

1. Please indicate your current level of satisfaction with the below aspects of information provision regarding introduction of opt out legislation. *

Completely unsatisfied

Somewhat unsatisfied

Neither satisfied nor unsatisfied

Somewhat satisfied

Completely satisfied

For the information I have received regarding general implications of the legislation changes, I am...

For the information I have received regarding specific implications of the legislation changes for my service, I am...

1. Do you have any further comments regarding the information you have received, or outstanding information needs in relation to the new legislation (opt out)?

(if none please enter 'none')

1. Please indicate your response to the following *

Not at all effective

Not very effective

Somewhat effective

Highly effective

Unsure how effective

Overall, how effective do you believe the new

opt out system will be in increasing supply of

eye tissue?

1. Do you have any other comments regarding the move to opt out legislation governing tissue and organ donation in England, with respect to donation of eye tissue?

(if none, please enter 'none')

# Preferences

The following questions are about provision and needs relating to knowledge and training about eye donation.

1. Have you received any in-service training about eye donation? *

Yes

No

1. Who provided the training? *

My employer (e.g. hospital or hospice)

Other

1. How long ago did you undertake the training? *

In the last 6 months

6-12 months ago

13-24 months ago

More than 24 months ago

1. Did the training provide you with the information you needed to be confident in discussing eye donation with patients/family members? *

Yes

Somewhat

No

1. Do you have any further comments on the positive or negative aspects of the training and/or information provided? (if none, please enter 'none') *
2. If you feel that you have unmet knowledge or training needs relating to eye donation please describe these below

(if none, please enter 'none') *

1. Do you know where to find out information about eye donation if you need to? *

Yes

No

1. Please describe below the information currently available to you regarding eye donation? (e.g. policies and procedures, information for patients and families etc.) (if unsure, please write 'unsure') *
2. Please indicate below where this information about eye donation is held in your organisation

(if unsure, please write 'unsure') *

1. What further information would you like to have regarding eye donation? (if none, please write 'none') *

# Flash quiz (FQ)

1. How long after death can eye donation take place?
2. What are definite contraindications for eye donation?
3. Is there anything that needs to happen before eye donation can take place?
4. Of the following evidenced based barriers to eye donation in palliative and hospice settings, which do you view as being the most influential?

*Please rank your responses from 1 = least influential to 5 = most influential*

1 2 3 4 5

Health care providers are reluctant to discuss the option of eye donation due to concerns that they will cause distress

Health care providers lack knowledge in what needs to be done to organise eye donation

Patients and family members are unaware of eye donation as an end of life option

The option of eye donation is not

'embedded' as a routine part of end of

life care clinical practice

Personal attitudes toward eye donation undermine clinical guidance and local policy about raising this option

# What needs to change?

1. In your view what are the key barriers to eye donation? (if unsure, please enter 'unsure') *

# Thank you

We appreciate you taking the time to complete this survey and for adding to the knowledge base being generated by the EDiPPPP study [(https://www.southampton.ac.uk/healthsciences/research/projects/edipppp.page (https://www.southampton.ac.uk/healthsciences/research/projects/edipppp.page)](https://www.southampton.ac.uk/healthsciences/research/projects/edipppp.page))

For any questions about the survey please contact the study team: Dr Tracy Long-Sutehall (T.Long@soton.ac.uk)

Dr Mike Bracher (M.J.Bracher@soton.ac.uk)

Dr Banyana C Madi-Segwagwe (B.C.Madi-Segwagwe@soton.ac.uk) When you are ready to complete, please click the 'Submit' button below

This content is neither created nor endorsed by Microsoft. The data you submit will be sent to the form owner.

Microsoft Forms
